# Genome-wide examination of the transcriptional response to ecdysteroids 20-hydroxyecdysone and ponasterone A in *Drosophila melanogaster*

**DOI:** 10.1186/1471-2164-12-475

**Published:** 2011-09-29

**Authors:** Sarah E Gonsalves, Scott J Neal, Amy S Kehoe, J Timothy Westwood

**Affiliations:** 1 Department of Cell & Systems Biology, University of Toronto, Mississauga, Ontario, Canada; 2 Department of Biology, Brandeis University, Waltham, Massachusetts, USA; 3 Nematology Unit, Canadian Food Inspection Agency, Ottawa, Ontario, Canada

## Abstract

**Background:**

The 20-hydroxyecdysone (20E) hierarchy of gene activation serves as an attractive model system for studying the mode of steroid hormone regulated gene expression and development. Many structural analogs of 20E exist in nature and among them the plant-derived ponasterone A (PoA) is the most potent. PoA has a higher affinity for the 20E nuclear receptor, composed of the ecysone receptor (EcR) and Ultraspiracle proteins, than 20E and a comparison of the genes regulated by these hormones has not been performed. Furthermore, in *Drosophila *different cell types elicit different morphological responses to 20E yet the cell type specificity of the 20E transcriptional response has not been examined on a genome-wide scale. We aim to characterize the transcriptional response to 20E and PoA in *Drosophila *Kc cells and to 20E in salivary glands and provide a robust comparison of genes involved in each response.

**Results:**

Our genome-wide microarray analysis of Kc167 cells treated with 20E or PoA revealed that far more genes are regulated by PoA than by 20E (256 vs 148 respectively) and that there is very little overlap between the transcriptional responses to each hormone. Interestingly, genes induced by 20E relative to PoA are enriched in functions related to development. We also find that many genes regulated by 20E in Kc167 cells are not regulated by 20E in salivary glands of wandering 3^rd ^instar larvae and we show that 20E-induced levels of *EcR *isoforms *EcR-RA, ER-RC*, and *EcR-RD/E *differ between Kc cells and salivary glands suggesting a possible cause for the observed differences in 20E-regulated gene transcription between the two cell types.

**Conclusions:**

We report significant differences in the transcriptional responses of 20E and PoA, two steroid hormones that differ by only a single hydroxyl group. We also provide evidence that suggests that PoA induced death of non-adapted insects may be related to PoA regulating different set of genes when compared to 20E. In addition, we reveal large differences between Kc cells and salivary glands with regard to their genome-wide transcriptional response to 20E and show that the level of induction of certain EcR isoforms differ between Kc cells and salivary glands. We hypothesize that the differences in the transcriptional response may in part be due to differences in the EcR isoforms present in different cell types.

## Background

In *Drosophila *and other arthropods, pulses of the steroid hormone 20-hydroxyecdysone (20E) are responsible for the temporal coordination of larval molts and metamorphosis. Physiological responses during these events can be diverse; for example during metamorphosis obsolete larval tissues are destroyed and adult structures arise from imaginal disc cells. Remarkably, these actions are carried out in a coordinated, tissue specific manner. At the site of target tissues, 20E binds to its cognate nuclear receptor triggering a cascade of gene activation. Primary 20E-inducible genes, which are directly induced by the steroid-receptor complex, are the earliest genes in the cascade to be transcribed and are insensitive to protein synthesis inhibitors. In contrast, secondary 20E-inducible genes are expressed later and are dependent on the synthesis of primary-response genes. Early studies of the 20E cascade looking at the puffing patterns of polytene chromosomes of late 3^rd ^instar larvae in response to 20E, predicted that primary-response genes would code for proteins that are responsible both for the induction of secondary response genes as well as for the inhibition of their own transcription [1]. Three of the most well characterized 20E-primary response genes, *Eip74EF, Eip75B*, and *br *fit this description perfectly. Furthermore, these three genes reside at chromosome cytolocations 74EF, 75B, and 2B5 respectively, which, along with approximately three other loci, exhibit rapid and dramatic puffing after exposure to 20E either naturally or artificially [2-4]. Likewise, the earliest characterized secondary response genes are found in a region (71E) that forms a distinct yet delayed puff upon 20E exposure [5,6]. Although the precise function of these genes has yet to be determined, based on their genomic sequence they are thought to encode effector proteins which is consistent with early predictions of secondary-response gene function [5]. Since the original characterization of these 20E-response genes, however, many examples of primary and secondary response genes with diverse functions have emerged, and many of these 20E-inducible genes do not appear to be associated with any identifiable 20E-induced puffs [7-13] underling the importance identifying individual components of the cascade for a clearer picture of 20E action.

The transcription factor complex coordinating the entire 20E-hierarchy, the 20E nuclear receptor heterodimer is composed of the vertebrate retinoid X receptor homolog, Ultraspiracle (USP) and the ecdysone receptor (EcR). EcR isoform distribution differs between tissues and typically those tissues expressing different isoforms show different responses to ecdysone at metamorphosis [14-17]. For example, immunolocalization experiments examining EcR isoform expression pattern at the onset of metamorphosis show that larval tissues destined to die during metamorphosis, such as the salivary gland, stain strongly for isoform B1 and weakly for isoform A whereas proliferating tissues, such as imaginal discs, show a reciprocal pattern staining strongly for isoform A and weakly for isoform B1 [16]. The most recent annotation of the *Drosophila *genome documents five EcR isoforms that differ in sequence at the amino terminus but share common DNA- and ligand- binding domains. The ligand-binding pocket of the 20E receptor shows remarkable flexibility enabling its activation by a variety of steroidal and non-steroidal 20E analogs/agonists [18]. For example, the ligand-binding domain of the *Drosophila *EcR homolog in *Heliothis virescens *adopts different structures in the presence of different 20E analogs [18].

Of the insect and plant derived analogs characterized, ponsterone A (PoA) is the most potent agonist of the EcR receptor [19] with an affinity approximately eight times greater than 20E [20]. As such, PoA, which differs from 20E by the abscence of a single hydroxyl group, has been used to work out numerous kinetic and physical parameters of the EcR [21]. Interestingly, the natural role of this and other phytoecdysteroids is still under debate, although most evidence disfavors a hormonal role in plants [22]. Instead, it is predicted that phytoecdysteroids induce precocious molting and subsequently death in insects providing an effective defense against insect feeding [22]. Given the increased binding affinity of PoA over 20E as well as its potential use in insect control, it is of importance to understand how this phytoecdysteroid affects transcription on a global scale.

The *Drosophila *Kc cell line is one of the most well documented 20E-responsive cell lines currently available and has been used in numerous studies examining the effects of 20E. In this study we use cDNA-based microarrays representing approximately 80% of the *Drosophila *genome to identify 20E-responsive genes in Kc167 cells. By exploiting the sensitivity of secondary-response gene transcription to protein synthesis, we are able to determine which of these genes are primary and which are secondary 20E-inducible genes. Furthermore, the transcriptional response to physiological levels of 20E is compared to that elicited under two other conditions: a 20-fold higher concentration of 20E or its plant derived structural analog, PoA. These analyses lead to the identification of 35 genes that reacted similarly to all three treatments. In addition to examining the transcriptional response to 20E in Kc cells, we were also interested in examining the response in a natural target tissue of 20E signaling, salivary glands. Salivary glands have been critical in the elucidation of the 20E signaling hierarchy through the examination of puff patterns on polytene chromosomes. By focusing on the response of the salivary gland in the isolation from other larval tissues we were able to identify many 20E-responsive genes that were not detected when whole larvae were examined [13]. Taken together, this work provides a detailed picture of the genes involved in the ecdysone hierarchy of gene transcription in Kc167 cells and salivary glands.

## Results and Discussion

### Identification of 20-hydroxyecdysone (20E)-responsive genes in Kc167 cells

The 20E-hierarchy of gene transcription serves as a good model for examining hormonal control of development. To gain insight into those genes that are part of the hierarchy, we assayed for 20E-induced changes in gene transcription across the genome. In the first part of this study, the transcriptional response to 0.5 μM 20E was examined in the *Drosophila *cell line, Kc167. Because 0.5 μM 20E causes the polytene chromosome puffs at the 74EF and 75B early 20E-inducible loci to reach their maximum size after 4 hours of organ culture [2] and *in situ *hybridization using labeled RNA produced after 20E treatment shows puff specific RNAs also increase along with puff size [23,24] the transcriptional response was examined after a 4 hour treatment with 0.5 μM 20E. The transcriptional profile of cells exposed to 0.5 μM 20E for 2 hours was also examined to identify any genes that may be induced early on but turned off after 4 hours. To allow identification of 20E-responsive genes in a controlled extracellular environment, in the absence of endogenous hormones, these experiments were conducted *in vitro*. Kc167 cells were used because the Kc cell line is responsive to 20E both morphologically [25-27] and transcriptionally [28] and is commonly used for the study of 20E. To examine the transcriptional response to 20E on genome-wide scale, RNA was isolated from both 20E-treated cells and untreated control cells, reverse-transcribed and labelled with Cy5 or Cy3 dye-coupled nucleotides respectively, and co-hybridized to cDNA microarrays. Following data acquisition and analysis (described in the Methods section), transcripts with at least a 1.5 fold change in abundance in response to 20E were identified. After a 2 hour treatment with 20E, 27 genes were induced (i.e. had increased transcript levels) (see Additional file 1: 20E-responsive genes in Kc167 cells after 2 and 4 hours of exposure to 0.5 μM 20E). Two-thirds of these genes (18) including the known 20E-inducible genes *Eip75, Br, Eip28/29*, and *Eip55*, remained significantly up-regulated after 4 hours in the presence of 20E while 51 additional genes were induced and 77 genes were repressed (i.e. had decreased transcript levels) (see Additional file 1: 20E-responsive genes in Kc167 cells after 2 and 4 hours of exposure to 0.5 μM 20E). Among the 20E-induced genes indentified here in Kc cells, the most strongly induced after 4 hours, *Eip28/29*, was originally identified owing to its quick reaction to 20E in Kc cells [29,30] and has since been used to study aspects of the 20E response such as its tissue specificity [31]. Taken together, the response observed here after a 2 and 4 hour 20E-treatment are consistent with two prior observations on the 20E-induced puffing patterns of larval salivary glands: first, that primary 20E-response genes are likely to still be detectable after a four-hour 20E-treatment and second, that the 20E-response of Kc167 is more robust after 4 hours of 20E exposure [4] (see Additional file 1: 20E-responsive genes in Kc167 cells after 2 and 4 hours of exposure to 0.5 μM 20E).

Of the 20E-inducible genes identified here, 30% were also identified in a recent study by Gauhar and colleagues that examined the transcriptional response of Kc cells following either a 1, 3, or 6 hr 20E treatment [32]. However, if we apply more stringent fold-change criteria to the list of 20E indentified here the degree of overlap increases such that at a 2-fold cut-off, there is 50% overlap, and there is 100% overlap between ours and the Gauhar et al. lists if we select a 3-fold change in expression. Remaining differences in the identification of 20E responsive genes indentified is likely due in part to the use of different culturing medium as we have previously found that media composition affects the transcriptional response of Kc cells to 20E (unpublished results). The use of a different microarray platform in the measurement of the transcriptional profile could also be a contributing factor.

### Identification of primary 20E-response genes

Having identified genes involved in the 20E-response, we were interested in determining where these genes fit into the 20E-hierarchy of gene activation. In particular, we wanted to identify those genes comprising the primary response to the hormone and are thus likely to be directly induced by 20E. Maximum induction of primary-response genes by 20E is expected to occur after 4 hours of exposure to the hormone, however, some secondary-response gene transcription may have already begun at this point [1,4]. One distinguishing characteristic of primary and secondary-response genes is their dependence on protein synthesis for transcription; only primary response genes are transcribed in the absence of protein synthesis [1,33]. Thus, to identify primary response genes, cells were treated with 20E in conjunction with an inhibitor of protein synthesis, cycloheximide (Table 1). RNA isolated from treated cells was labelled and co-hybridized to cDNA arrays along with a differentially labelled untreated control. From this analysis we identified 149 genes that were induced and 119 genes that were repressed by the 20E-cycloheximide combination (data not shown). To determine which of these genes were induced/repressed in response to 20E, and were not responding to exposure to cycloheximide alone, additional microarrays were run using RNA from Kc167 cells treated only with cycloheximide. From this experiment, 214 genes were indentified whose transcription is altered due solely to the inhibition of protein synthesis (data not shown). A two-class SAM analysis identified 35 genes whose transcripts levels in the presence of cycloheximide differs significantly when 20E is present including the well characterized 20E-primary-response genes, *Eip75B *and *br *(Table 1 and Additional file 2: Extended version of Table 1). Although *Eip74EF *was not among the genes on this list, we confirmed its induction by 20E by qRT-PCR (see Additional file 3: qRT-PCR confirmation of microarray data) suggesting that the cDNA probe on the batch of arrays used for this study was likely of poor quality and unable to detect transcripts of this gene. Of the 35 primary-response genes identified, 15 were not induced by 20E alone. The induction of these 15 genes by 20E is likely masked in the absence of cycloheximide due to a negatively regulated auto-feedback loop where primary-response gene transcription is repressed by primary-response gene protein products [1]. Interestingly, the only gene transcript repressed as part of the primary response to 20E, *CG3752*, also evades detection unless protein synthesis is blocked (Table 1). The majority of 20E-responsive genes identified after a 4 hour treatment with 0.5 μM 20E behaved as secondary-response genes as their response to 20E was blocked in the presence of cycloheximide; 48 genes were induced and 77 were repressed as part of the secondary response (Table 1 and Additional file 2: Extended version of Table 1).

**Table 1:** Primary and secondary response 20E-regulated genes identified in Kc167 cells

Group	Gene Symbol	20E Fold Difference	q value (%)	Primary fold difference	q value (%)	Select Functional Annotation Terms Enriched in Group
	*br*	3.58	0.00	4.75	0.00	
		
	*CG5346*	3.10	0.00	2.91	0.00	induction of
		
	*Rrp46*	2.45	0.00	2.68	0.00	programmed cell
		
	*Pect*	2.89	0.00	2.49	0.00	death by hormones;
		
	*CG14523*	1.83	0.40	2.37	0.00	
		
	*Cyp9c1*	1.87	1.13	2.35	0.00	catalytic activity;
		
	*granny-smith*	1.79	0.00	2.21	0.00	
		
	*CG4825*	2.10	0.00	2.14	0.00	cytochrome P450,
		
	*CG17760*	2.51	0.00	2.13	0.00	E-class, group I;
		
E	*Eip55E*	1.92	0.00	2.03	0.00	
		
	*CG15482*	2.75	0.00	1.83	0.00	hydrolase activity;
		
	*Eip75B*	1.82	0.00	1.83	0.00	
		
	*Nc*	1.40	0.00	1.80	0.00	
		
	*eater*	1.97	0.00	1.76	0.00	
		
	*Idgf2*	-1.06		1.74	0.00	
		
	*CG11586*	1.00		1.74	1.89	
		
	*PRL-1*	2.10	0.00	1.70	0.00	
		
	*l(2)09851*	-1.08		1.70	0.00	
		
	*vri*	1.52	0.84	1.68	0.00	
		
	*ImpL2*	1.42		1.68	0.00	
						

	*Eip71CD*	4.06	0.00			
		
	*CG11893*	2.28	0.62			mitochondrial
		
	*CG15711*	2.20	0.00			respiratory chain;
		
	*CG30104*	2.03	0.00			
		
F	*CG5059*	2.00	0.34			glutathione
		
	*CG17819*	2.00	0.72			S-transferase;
		
	*CG5104*	1.99	0.00			
		
	*CG31633*	1.89	2.76			cellular metabolic
		
	*CG5694*	1.87	4.45			process;
		
	*Obp8a*	1.83	3.56			
						

G	*Aldh*	-1.32	3.56	-2.05	2.10	
						

	*CG8801*	-1.84	0.62			
		
	*ncd*	-1.92	0.38			nucleotie-binding;
		
	*Hsp70Aa*	-2.01	0.49			
		
	*Hsp68*	-2.13	1.13			kinase activity;
		
H	*Scp2*	-2.13	4.45			
		
	*gammaTub23C*	-2.17	1.13			glycolysis;
		
	*Hsp60*	-2.43	0.00			
		
	*Acon*	-2.51	1.76			cytoskeleton
		
	*Vago*	-2.57	0.00			organization and
		
	*Hsp70Ab*	-2.91	1.13			biogenesis

A two- class SAM analysis of triplicate data identified primary-response genes that responded to 20E in the presence of cycloheximide. The top 20 up-regulated primary-response genes and single down-regulated primary-response gene are listed (group E and G respectively). Secondary-response genes regulated by 20E only in the absence of cycloheximide were also identified. The top 10 up- and down-regulated secondary-response genes are listed (groups F and H respectively). The fold change of each gene following a 4 hour treatment with 0.5 μM 20E (20E fold difference) and, for primary-response genes, the fold change following a 4 hour treatment with 0.5 μM 20E and 100 μM cycloheximide (primary fold difference) is given. q-values determined by SAM represent the lowest false discovery rate at which that gene is considered significant. For a gene to be included as differentially expressed, it must have a q-value < 5% in that experimental condition. q-values > 5% are not shown. DAVID (http://david.abcc.ncifcrf.gov/) was used to identify enriched annotation categories among groups of genes that respond similarly to 20E. Some of the most enriched functional terms associated with each group (i.e. terms with modified Fisher Exact p-values ≤ 0.05; where the p-value gives the probability of that term being randomly associated with the genes in the group) are given in the right most column. A complete list of all genes in these groups and their functional annotation is in Additional file 2.

In addition to differences in transcriptional response in the absence of protein synthesis, it is also expected that most primary-response genes encode regulators while secondary-response genes encode effectors [1,5,7,10]. To determine if this is the case for the genes identified here as part of the primary and secondary response, we examined each group of genes for enrichment in functional annotation terms/keywords using the online resource DAVID. The results of this analysis are summarized in Table 1. Several of the primary-response gene transcripts include those that code for the well known DNA binding transcription factors *Eip75B *and *br *as well as *vrille *which is consistent with the model that primary-response proteins are required for the transcriptional induction of the secondary-response genes. "Induction of programmed cell death by hormones" is among the most highly enriched terms associated with primary-response genes and comes as no surprise due to the well established involvement of 20E-regualted genes such as *br *and *Nc *in cell death pathways during *Drosophila *development [34-37]. However, we should point out that that Kc cells do not undergo apoptotic cell death in response to ecdysone treatment and therefore other factors and/or conditions must need to be present in order to carry out this particular process. "Catalytic activity" and "hydrolase activity" were both also among the most significantly enriched terms supporting the idea that primary-response genes are involved in a broad range of regulatory roles [7-13]. For example, one of the genes belonging to the hydrolase class includes imaginal disk growth factor 2, a protein that when bound to its receptor initiates signal transduction cascades important to imaginal disk development. Some primary- and secondary-response genes include genes that code for proteins involved in cell movement and organization and/or are associated with the cytoskeleton. These include up-regulated transcripts for *Roadblock*, a gene coding for a dynein-associated protein and down-regulated transcripts for *Spastin*, and a gene coding for a microtubule severing protein. Transcript changes in genes of this sort are consistent with the cell movement and morphogenetic changes that occur during 20E-dependent developmental changes. Also of interest is that several of the secondary-response genes include genes that are involved in metabolic processes. More specifically, there seems to be an increase in transcripts for genes involved in mitochondrial respiration and a decrease in transcripts for genes involved in glycolysis perhaps suggesting that in addition to the cellular organization and tissue changes that are induced by 20E, there is also a shift in how energy is being produced.

### Transcriptional response of Kc167 cells to an increased concentration of 20E and to its structural analog, ponasterone A (PoA)

To examine the specificity of the 20E-response of Kc167 cells to both hormone concentration and ligand structure, two other conditions were tested: a 20-fold higher concentration of 20E, 10 μM, and the replacement of 20E with its structural analog, PoA. 20E concentration is known to affect at least three aspects of the 20E-response: the size of both early and late puffs [2], the rate of early puff regression [2], and the transcription of some early gene isoforms [38]. PoA, often used as substitute for 20E, is a more potent activator of the 20E-response. At least four different measures indicate that PoA activity is approximately eight fold higher than 20E activity: affinity for the ecdysone receptor [20], morphological changes associated with the 20E-response [25], the level of induction of known 20E-inducible proteins [29], and the level of transcription from an ecdysone response element (EcRE) [19]. Thus, to minimize effects caused by the difference in receptor affinity and make the comparison between analogs as similar as possible, our working concentration of PoA was eight fold less than that of 20E (ie. 0.0625 μM PoA versus 0.5 μM 20E).

Genes that responded to either of the two treatments were identified by microarray analysis as described above. Compared to the number of genes induced by a 4 hour treatment with 0.5 μM 20E, both 10 μM 20E and 0.0625 μM PoA stimulated the transcription of a greater number of genes: 85 and 115 respectively versus 69 induced by 0.5 μM 20E (see Additional file 4: Genes identified as part of the transcriptional response to either 20E or PoA). Furthermore, PoA down-regulated substantially more genes than either 20E treatment. One-hundred and fifty genes were repressed after a 4 hour treatment with PoA - almost double the number repressed by 0.5 μM 20E (79) and nearly six fold more than the number repressed by 10 μM 20E (26) (see Additional file 4: Genes identified as part of the transcriptional response to either 20E or PoA). It is possible that the differences in the response to PoA may be due to the activation of additional nuclear receptors; it is only assumed to be the 20E receptor heterodimer because it has been shown to be the case *in vitro *[22].

**Figure 1:**
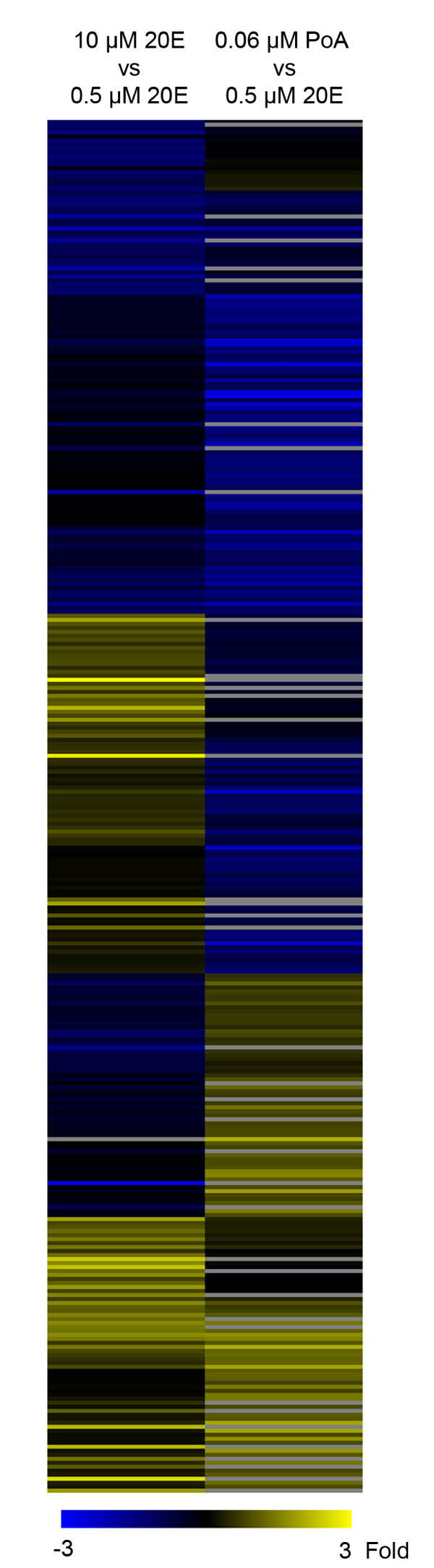
**Effect of PoA and increased 20E concentration on gene regulation**. A multi- class SAM analysis identified groups of genes that are differentially regulated by either 10 μM 20E and/or 0.0625 μM PoA relative to 0.5 μM 20E from a list of genes exhibiting a +/- 1.5 fold change in response to at least one of the two treatments. Genes showing a transcriptional response to either 10 μM 20E or 0.0625 μM PoA that significantly differed from its response to 0.5 μM 20E were clustered based on fold difference in expression relative to its response to 0.5 μM 20E. Grey represents missing data.

Overall, 379 genes were identified that responded to at least one of the three hormone treatments examined (0.5 μM and 10 μM 20E and 0.0625 μM PoA; see Additional file 4: Genes identified as part of the transcriptional response to either 20E or PoA). To determine which of these 379 hormone-responsive genes were sensitive to all three conditions tested and which are differentially regulated by either 10 μM 20E or PoA relative to 0.5 μM 20E, a multi-class SAM analysis was performed where the transcriptional response to 10 μM 20E and PoA was compared to the transcriptional response 0.5 μM 20E for each gene. This analysis revealed two things about the transcriptional response to each of the hormone conditions tested. First, of the 379 genes that responded to at least one of the three treatments, 35 showed no significant difference in their transcriptional response to all three treatments and eight of these are primary 20E-inducible genes (Table 2). Second, PoA produced a more distinct transcriptional profile than did an elevated concentration of 20E with respect to the 0.5 μM 20E treatment (Figure 1), suggesting that PoA may not be acting solely through the ecdysone hierarchy. We confirmed the transcriptional response of three key 20E-inducible genes, the ecdysone-receptor complex, and a novel 20E-incudible gene by qRT-PCR. In all cases, the transcriptional response for these genes measured by qRT-PCR, although differing in magnitude when compared to the microarray analysis (i.e. showing on average a 5.58 fold more induction than the microarray data), had the same direction of change (see Additional file 3: qRT-PCR confirmation of microarray results).

**Table 2:** Genes that respond similarly to 0.5 μM 20E, 10 μM 20E, or 0.0625 μM PoA

Gene Symbol or Clone ID	0.5 μM 20E Fold Difference	q value (%)	10 μM 20E Fold Difference	q value (%)	0.0625 μM PoA Fold Difference	q value (%)	GO Molecular Function
*Eip71CD*	4.07	0.00	3.58	0.00	3.51	0.00	oxidoreductase activity

***CG5346***	**3.10**	**0.00**	**2.88**	**0.00**	**3.49**	**0.00**	**cation binding**

***Pect***	**2.88**	**0.00**	**2.91**	**0.00**	**2.49**	**0.00**	**transferase activity**

***PRL-1***	**2.10**	**0.00**	**2.12**	**0.00**	**1.58**	**0.00**	**hydrolase activity**

***CG4825***	**2.10**	**0.00**	**2.17**	**0.00**	**2.56**	**0.00**	**transferase activity**

*CG30104*	2.03	0.00	2.40	0.00	2.39		hydrolase activity

***eater***	**1.97**	**0.00**	**2.52**	**0.00**	**2.20**	**0.00**	**receptor binding**

***Cyp4e2***	**1.83**	**0.00**	**2.05**	**0.00**	**2.02**	**0.00**	**monooxygenase activity**

***CG14523***	**1.83**	**0.40**	**2.04**	**0.00**	**1.81**	**0.00**	**peptidase activity**

***granny-smith***	**1.79**	**0.00**	**1.79**	**0.00**	**1.94**	**0.00**	**cation binding**

*CG5958*	1.53	0.40	1.65	0.00	1.82	0.00	retinal binding

*RE28720*	1.52	3.56	1.67	0.00	1.54	0.00	N/A

*RH68619*	1.49	0.00	1.75	0.00	1.68	0.00	N/A

*mTerf3*	1.46	4.45	1.69	0.00	1.70	0.00	protein binding

*CG33969*	1.40	0.84	1.50	0.00	1.19	0.73	N/A

*Ggamma1*	1.39		1.27	0.47	1.58	0.11	hydrolase activity

*CG8507*	1.38		1.18	4.43	1.57	0.00	low-density lipoprotein receptor binding

*CG18591*	1.35		1.26	0.47	1.62	0.16	RNA binding

*HL03650*	1.33	1.50	1.54	0.00	1.74	0.00	N/A

*GstE1*	1.05		-1.01		-1.52	0.00	transferase activity

*RE45701*	-1.22	2.76	-1.65	0.47	-1.56	0.00	N/A

*CG7706*	-1.22		-1.62	0.63			anion transporter activity

*vas*	-1.27		-1.52	0.00			ATP-dependent helicase activity

*CG14739*	-1.28	0.62	-1.53	0.63			ubiquitin conjugating enzyme activity

*Pde8*	-1.30		-1.49	0.00	-1.58	0.00	hydrolase activity

*LD33681*	-1.35		-1.31	0.00	-1.67	0.00	N/A

*CG13868*	-1.36	0.49	-1.35	0.30	-1.58	0.00	protein binding

*Gs1*	-1.39	2.17	-1.58	0.00	-1.07		ligase activity

*arm*	-1.39	3.56	-1.59	0.00	-1.30	0.14	alpha-catenin binding

*Rpn1*	-1.41		-1.15		-1.54	0.49	peptidase activity

*CG10641*	-1.47	0.00	-1.34	1.60	-1.58	0.00	cation binding

*eIF3-S10*	-1.55	0.00	-1.36	0.00	-1.75	0.28	translation factor activity

*RhoGAP16F*	-1.65	0.00	-1.34	0.00	-1.59	0.00	GTPase activator activity

*RH07164*	-1.86	4.45	-1.59	0.30	-1.86	0.14	N/A

A multi-class SAM analysis revealed genes with similar transcriptional responses to the hormones and hormone concentrations studied. Genes identified as primary 20E-response genes (see Table 1) are indicated in bold. As with Table 1, the fold change and q-value of each gene following the treatment indicated is given. A missing fold change value means that gene was either not represented by at least 2/3 spots and/or did not exhibit a coefficient of variation of at least 1 for that treatment across replicate arrays.

Given the observed similarities and differences in the ecdysteroid-response to both an increased 20E-concentration and the use of a structural analog, we wanted to identify functions associated with genes that responded similarly to all treatments tested. Using DAVID we found two categories enriched in similarly responding genes that were also enriched among primary-response genes: "catalytic activity" and "hydrolase activity" (Table 3). Conversely, genes that reacted significantly different to either 10 μM 20E, PoA or 0.5 μM 20E were enriched in many unique categories; the most strongly enriched functional terms are associated with genes down-regulated by PoA relative to 0.5 μM 20E (Table 3). Furthermore, other categories related to development are also strongly enriched in the list of genes that are down-regulated by PoA relative to 0.5 μM 20E such as "anatomical structure development", "induction of programmed cell death", and "instar larval or pupal development" (Table 3 and Figure 1). The apparent lack of activation of developmental genes by PoA, as indicated by this analysis, is suggestive of an alternate mode of action of PoA induced death in non-adapted insects more so than the simple induction of premature molting via the canonical ecdysone hierarchy. In fact, the results potentially suggest that PoA might interfere with normal 20E induced transcriptional changes and prevent the induction of some of the genes that are required for 20E dependent developmental processes.

**Table 3:** Annotation enrichment of genes regulated by either 0.5 μM or 10 μM 20E or 0.0625 μM PoA

Group	Response	# Genes in Group with Valid Identifier/Total # in Group	Category	Term	p-value	Number of Genes from Group Annotated with Term
			MF	catalytic activity	9.35E-03	18
			
			BP	positive regulation of translation	2.84E-02	2
			
1	Common to all three conditions tested	30/35	BP	protein metabolic process	2.84E-02	12
			
			BP	primary metabolic process	2.96E-02	19
			
			MF	hydrolase activity	3.81E-02	10

			BP	response to temperature stimulus	4.16E-03	4
			
2	Induced by both PoA &10 μM 20E	54/58	SP	DNA binding	1.40E-02	7
			
			BP	lipid transport	2.87E-02	3

			CC	intracellular part	1.34E-02	21
			
3	Repressed by both PoA & 10 μM 20E	46/54	BP	protein folding	1.99E-02	4
			
			BP	generation of precursor metabolites and energy	2.94E-02	6
			
			CC	mitochondrial respiatory chain	3.63E-02	3

			CC	organelle part	6.17E-04	21
			
			CC	organelle membrane	3.47E-03	9
			
4	Induced by PoA	87/89	CC	cytoplasm	4.16E-03	22
			
			BP	intracellular protein transport	1.08E-02	8
			
			SP	ribonucleoprotein	1.17E-02	5
			
			BP	cytoplasmic sequestering of transcription factor	2.55E-02	2

			BP	developmental process	2.39E-06	48
			
			MF	nucleoside-triphosphatase activity	1.84E-05	20
			
			BP	cell differentiation	1.86E-05	31
			
			MF	hydrolase activity, acting on acid anhydrides	2.84E-05	20
			
			BP	gamete generation	3.68E-05	20
			
			BP	sexual reproduction	5.17E-05	20
			
			BP	anatomical structure development	6.51E-05	36
			
5	Repressed by PoA	133/145	BP	organelle organization and biogenesis	1.98E-04	27
			
			BP	neuron development	2.00E-04	13
			
			BP	system development	2.08E-04	31
			
			MF	helicase activity	9.13E-04	8
			
			BP	induction of programmed cell death	1.16E-03	6
			
			MF	ATPase activity	1.47E-03	13
			
			BP	instar larval or pupal development	2.10E-03	16
			
			BP	organ development	3.44E-03	24
			
			BP	immune system development	4.29E-03	6
			
			BP	DNA packaging	9.87E-03	8
			
			BP	response to ecdysone	3.73E-02	3

Expression differences are relative to the 0.5 μM 20E dataset (i.e. "induced by PoA" indicates genes that were more greatly induced by 0.0625 μM PoA than by 0.5 μM 20E). DAVID (http://david.abcc.ncifcrf.gov/) was used to identify enriched annotation categories among groups of genes that responded similarly to one or more of the hormone treatments examined in this study (see Additional file 4: Genes identified as part of the transcriptional response to either 20E or PoA). The modified Fisher Exact p-value represents the probability that the number of genes associated with an annotation term in the sample list is random compared to the occurrence of the term in the all of the genes on the array used for the analysis (12kv1)). (BP) biological process; (MF) molecular function; (CC) cellular component; (SP) Swiss Prot Keywords; (INT) Interpro Name; (UP) UniProt Sequence Feature.

### Transcriptional response of salivary glands to 20E

**Figure 2:**
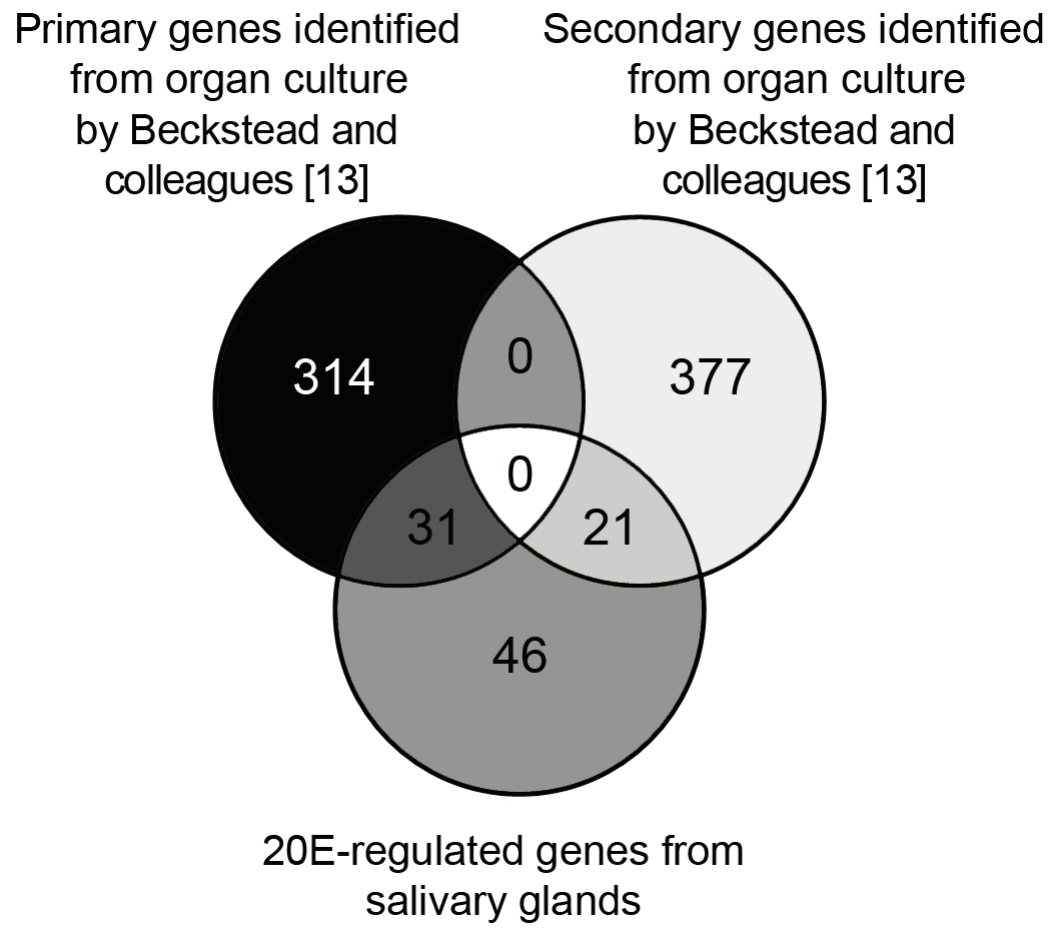
**Overlap of genes regulated by 20E in salivary glands and whole organ culture**. Venn diagram depicting overlap between number of genes identified as part of the 20E response in salivary glands and genes identified by Beckstead and co-workers [13] in whole organ culture as part of the primary or secondary response to 20E.

We wanted to extend our analysis to a 20E-responsive larval tissue to see how it compares to the 20E transcriptional response of Kc167 cells. Salivary glands were chosen in this respect due to the relative ease of dissection and because the foundations of our understanding of the 20E response comes from the study of salivary glands. Cultured glands from 20 late third instar larvae staged by the blue gut method (see Methods section) were treated with 0.5 μM 20E for four hours. We chose to treat salivary glands prior to the natural 20E pulse rather than assay glands after the natural pulse to rule out potential confounding effects caused by other endogenous hormones and/or the presence of protein products of secondary-response genes within the 20E hierarchy. RNA was isolated from 20E treated glands and untreated control glands, amplified, and analyzed by microarray experiments as described in the Methods section. We identified 98 up-regulated genes including several known 20E-inducible genes such as, *Eip75B, Eip74EF, EcR, Nc*, and *ImpL2*, and 8 down-regulated genes (see Additional file 4: Genes identified as part of the transcriptional response to either 20E or PoA). In a study by Beckstead and colleagues looking at 20E responsive genes in cultured larval organs 743 genes were identified as 20E responsive [13]. Their treatments, however, utilized twice the concentration of 20E and lasted for an additional two hours. Nevertheless, we were still able to identify 52 genes in common, the majority of which showed similar changes in expression level (Figure 2 and Additional file 5: Genes that respond to 20E in salivary glands and in *Drosophila *organ culture). By focusing only on salivary glands in this study we were able to identify 46 salivary gland specific 20E-inducible genes that were not identified in total organ preparations [13] (Figure 2 and Additional file 6: Salivary gland-specific 20E responsive genes). A DAVID analysis reveals that these salivary gland specific genes are enriched for protein binding (data not shown).

**Figure 3:**
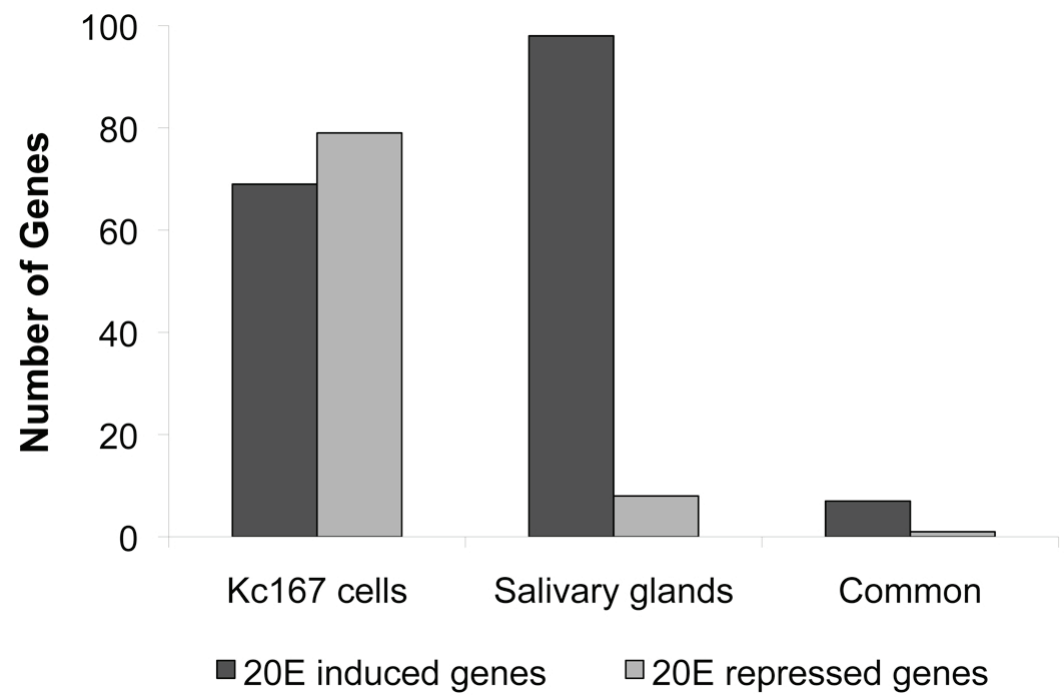
**The transcriptional responses of Kc167 cells and salivary glands to 20E exhibit very little overlap**. Roughly equal numbers of genes are induced (black bars) and repressed (grey bars) in cells by 20E. In salivary glands, the response to 20E is largely stimulatory with nearly 10 times more genes being induced as repressed. Only a small number of genes are regulated by 20E in both cells and glands (common).

Genes induced by 20E in salivary glands but not in Kc167 cells were enriched in GO biological processes such as "salivary gland histolysis", "programmed cell death" and various "developmental" processes as might be expected. "Cellularization" and "transporter activity" were also among the highest scoring hits. Conversely, genes that were up regulated only in Kc cells showed enrichment for "metabolic process" and "oxidoreductase activity"- categories that were also enriched among secondary response genes in Kc cells suggesting that the secondary response genes differ between Kc cells and salivary glands (Table 4). Comparison of 20E responsive genes from both systems allowed the identification of 10 genes whose response is conserved across both systems suggesting that they might occupy key positions within the hierarchy (Figure 3). Among the most highly induced of these genes are two genes that have not been traditionally associated with the 20E response in *Drosophila, CG6579 *and *Rrp46 *(see Additional file 4: Genes identified as part of the transcriptional response to either 20E or PoA). CG6579 is currently un-annotated and Rrp46 is a component of the exosome that has been shown to relocate to developmental loci during periods of active transcription and is involved in mRNA processing [39]. *Rrp46 *showed the greatest correlation in expression profile to *Eip75B *highlighting the importance of gene transcription in the ecdysone response.

**Table 4:** Annotation enrichment of genes that respond to 20E in either Kc167 cells or salivary glands

Response to 20E	# Genes in Group with Valid Identifier/Total # in Group	Category	Term	p-value	Number in Category	% of Total
		SP	chromoprotein	3.3E-03	3	5
		
		INT	peptidase M13	4.3E-03	3	5
		
		BP	electron transport	4.5E-03	7	13
		
Induced in Kc cells	56/69	BP	metabolic process	5.5E-03	31	55
		
		MF	oxidoreductase activity	1.0E-02	10	18
		
		INT	glutathione S-transferase	1.0E-02	3	5
		
		BP	generation of precursor metabolites and energy	1.6E-02	7	13
		
		MF	iron ion binding	3.1E-02	5	9

		BP	cellularization	2.4E-04	6	11
		
		BP	salivary gland histolysis	4.9E-04	6	11
		
		BP	programmed cell death	2.8E-03	9	16
		
		BP	cellular developmental process	3.4E-03	18	32
		
		BP	exocrine system development	3.4E-03	6	11
		
		BP	germ-band shortening	5.7E-03	3	5
		
		BP	gland development	6.6E-03	6	11
		
		BP	sperm individualization	1.5E-02	3	5
		
Induced in salivary glands	87/98	UP	compositionally biased region:Poly-Ala	1.4E-02	5	9
		
		MF	kinase activity	1.4E-02	9	16
		
		BP	regulation of metabolic process	1.5E-02	15	27
		
		MF	ligand-dependent nuclear receptor activity	2.8E-02	3	5
		
		BP	cell differentiation	1.7E-02	16	29
		
		BP	female meiosis chromosome segregation	3.2E-02	3	5
		
		MF	cytoskeletal protein binding	3.0E-02	7	13
		
		BP	embryonic development via syncytial blastoderm	4.3E-02	5	9
		
		BP	regulation of cellular metabolic process	4.8E-02	13	23
		INT	cell division/GTP binding protein	5.2E-02	2	4

		SP	nucleotide-binding	3.2E-08	20	28
		
		MF	kinase activity	6.2E-04	11	15
		
Repressed in Kc cells	71/77	INT	chaperonin Cpn60	5.7E-03	3	4
		
		MF	unfolded protein binding	1.2E-02	4	6
		
		SP	glycolysis	1.5E-02	3	4
		
		BP	cytoskeleton organization and biogenesis	2.5E-02	9	13

Repressed in salivary glands	9	N/A	N/A	N/A	N/A	N/A

DAVID (http://david.abcc.ncifcrf.gov/) was used to identify enriched annotation categories among groups of genes that respond similarly to 20E. The modified Fisher Exact p-value represents the probability that the number of genes associated with an annotation term in the sample list is random compared to the occurrence of the term in the all of the genes on the array used for the analysis (12kv1)). (BP) biological process; (MF) molecular function; (CC) cellular component; (SP) Swiss Prot Keywords; (INT) Interpro Name; (UP) UniProt Sequence Feature.

### Tissue specific differences in ecdysone receptor (EcR) isoform induction by 20E

Some of the differences in the transcriptional response of Kc cells and salivary glands may be explained by differential chromatin states of inactive genes in the two systems. For example, salivary gland secretion protein (*sgs*) genes known be under the control of the hierarchy were expressed in glands but not in cells most likely because of the difference in the two cell types. Differential gene regulation by 20E may also result from the expression of different *EcR *isoforms or combinations thereof [14-16,40]. Typically, tissues with dissimilar developmental fates express different *EcR *isoforms [14-16]. Our microarray results agree with previous findings that Kc cells express hemocyte and plasmatocyte marker genes *Pxn, ush*, and *Hml *[41] (data not shown) and that 20E treatment of Kc cells causes the induction of *Eip28/29 *and *Eip55 *(*Eip40*), both of which are 20E-regulated in the hematopoietic lymph gland [31] providing further support for an embryonic hemocyte origin of Kc cells [31,41]. In contrast to larval salivary glands, embryonic hemocytes persist in the adult fly [42] suggesting that Kc cells are likely more similar to imaginal disc or adult tissues than larval tissues and placing them in a different metamorphic class than the salivary gland [43].

**Figure 4:**
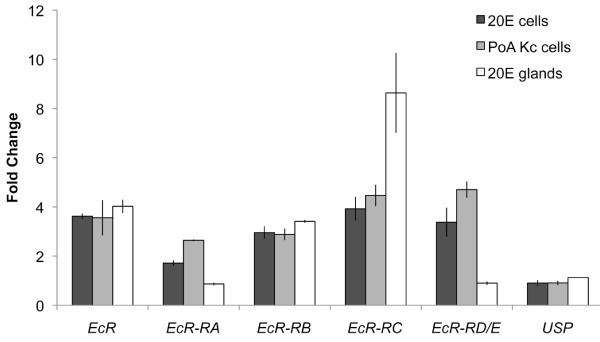
**The transcription of EcR isoforms are differentially regulated in Kc cells and salivary glands**. Relative induction of USP, EcR and five EcR isoforms in Kc cells and salivary glands by either 0.5 μM 20E or 0.0625 μM PoA as determined by qRT-PCR relative to untreated Kc cells or salivary glands, respectively.

Because *EcR *is a 20E-inducible gene, we first examined the overall level of induction of *EcR *by qRT-PCR using a primer designed against the 3' region of the gene common to all isoforms (Figure 4). Interestingly, unlike many of the 20E-inducible genes we identified by microarray analysis, *EcR *was induced to a similar extent in PoA-treated Kc cells, 20E-treated Kc cells and 20E-treated salivary glands (Figure 4). We next examined the transcriptional response of each *EcR *isoform to PoA and 20E using primers that recognize unique regions of each isoform and found that there was no difference in isoform regulation in Kc cells treated with either hormone (Figure 4). Comparison of isoform induction in 20E-treated Kc cells to 20E-treated glands, however, revealed a tissue specific pattern in isoform regulation (Figure 4). At least two *EcR *isoforms (*EcR-RA *and *EcR-RD *and/or *EcR-RE*; where *EcR-RD *and *EcR-RE *were indistinguishable by qRT-PCR analysis and will be referred to hereafter as *EcR-RD/E*) were induced in Kc cells by both PoA and 20E but not induced at all in salivary glands treated with 20E (Figure 4). It has previously been shown that the proteins encoded by the *EcR-RA, EcR-RD *and *EcR-RE *isoforms show strong expression in both embryos and imaginal discs [16] and EcR-A is thought to be responsible for adult differentiation [44]*EcR-RC*, on the other hand, although induced by both PoA and 20E in Kc cells, was more strongly induced by 20E in salivary glands (Figure 4).

Although differences in *EcR *isoform regulation in Kc cells and salivary glands by 20E may be linked to the variation in the observed transcriptional response to 20E, it is unlikely that the same explanation can be applied to account for the difference in response observed in Kc cells treated with 20E and PoA since qRT-PCR analysis reveals at best, subtle differences in *EcR *isoform regulation by 20E and PoA (Figure 4). It is tempting to speculate that the different EcR isoforms form 20E receptors with different target genes and thus contribute to differences in the 20E induced transcript profiles. Alternatively, the differences in transcript profiles may have very little to with which EcR isoforms are present and more to do with cell type differences of other proteins. For example, differences in the concentrations of particular chromatin proteins could affect the accessibility of the various target genes differently in different cell types. Also, the relative amount of proteins that partner with EcR to make the 20E heterodimer nuclear receptor could affect the sites the 20E receptor binds to. While the classical partner for EcR has been thought to be Ultraspiracle (USP), it is now known that biologically active 20E receptors can be made without USP [45]. In mid-third instar larvae, the 20E dependent induction of the glue proteins requires EcR but not USP suggesting that EcR makes 20E receptors that consist of EcR and yet to be characterized partner protein [45]. One approach to assess the relative contributions of each of the EcR isoforms would be to employ RNAi knockdown strategies of the different EcR isoforms in both Kc cells and larval tissues and examine what affect this has on the 20E induced transcript profiles.

## Conclusions

We have identified 35 primary 20E-response genes that are induced by 20E in the absence of protein synthesis in *Drosophila *Kc167 cells, one of the most widely used cell lines employed to study the ecdysone response. The primary 20E responsive genes were enriched for Gene Ontology (GO) terms such as induction of programmed cell death by hormones, catalytic activity, hydrolase activity, and cytochrome P450. We have also identified 125 secondary 20E-response genes (48 induced and 77 repressed). GO terms that are enriched in the secondary-response genes include an increase in transcripts for genes involved in mitochondrial respiration and a decrease in transcripts for genes involved in glycolysis suggesting that 20E induces shifts in cellular metabolism. Some primary- and secondary-response genes include genes that code for proteins involved in cell movement and organization and/or are cytoskeletal associated which is consistent with the cell movement and morphogenetic changes that occur during 20E-dependent developmental changes.

Comparison of the genome-wide transcriptional response to 20E to its plant derived structural analog ponasterone A (PoA) revealed a large difference in the transcriptional targets of these molecules. While these two compounds are structurally very similar, many more genes related to various aspects of development appear to be significantly induced by 20E than by PoA. More specifically, the most strongly enriched functional terms are associated with genes down-regulated (i.e. not induced) by PoA relative to 0.5 μM 20E. The genes not induced by PoA include GO terms such as developmental process, cell differentiation, gamete generation, anatomical structure development, organelle organization and biogenesis, neuron development, and induction of programmed cell death. The apparent lack of activation of developmental genes by PoA suggests that PoA induced death in non-adapted insects may be due to more than the just the simple induction of premature molting via the canonical ecdysone hierarchy.

We also compared the 20E response in Kc cells to that of a natural 20E target tissue where the function of 20E has been well described, the salivary glands of wandering 3^rd ^instar larvae, and found little overlap in 20E-responsive genes. Genes induced by 20E in salivary glands but not in Kc167 cells were enriched in GO biological processes such as "salivary gland histolysis", "programmed cell death" and various "developmental" processes as well as "cellularization" and "transporter activity".

To help identify a potential mechanism to explain the difference in the transcriptional responses in Kc cells and salivary glands, we analyzed the 20E-induced transcription of the various *EcR *isoforms, a known 20E-inducible gene and a component of the nuclear receptor complex that binds 20E. We did find differences in the induction of *EcR *isoforms *EcR-RA, ER-RC, and EcR-RD/E *between Kc cells and salivary glands in response to 20E. This suggests that the relative amount of various EcR isoforms present in a cell is different in different cell types and possibly contributes to the transcriptional response a given tissue has to 20E.

## Methods

### Hormone treatments

*Drosophila *Kc167 cells were obtained from *Drosophila *Genomic Resource Centre (Bloomington, IN) and were grown to confluence in Schneider's medium (Invitrogen) supplemented with 5% heat-inactivated FBS (Sigma) and 20 ug/ml gentamicin (Sigma). Cells were passaged into a series of new flasks that were divided into two groups, experimental and control, and were allowed to recover for one hour. Experimental cells were treated with 0.5 μM 20-hydroxyecdysone (20E) (Sigma) for two hours or with one of the following for four hours: 0.0625 μM ponasterone A (Sigma), 0.5 μM 20E, 10 μM 20E, 0.5 μM 20E plus 100 μM cycloheximide (Sigma), 100 μM cycloheximide alone, or with 0.05% or 0.5% ethanol (solvent used to dissolve hormones). All treatments were performed at 22°C and three independent biological replicates were assessed.

Late third instar larvae (*dp cn bw cl*) were selected by the blue gut method as previously described [46]. Salivary glands were dissected from 10 larvae in physiological saline solution and cultured in a 10-well dish with no more than five glands per well in 120 μl of modified TB1 buffer (15 mM HEPES, pH 6.8, 80 mM KCl, 16 mM NaCl, 5 mM MgCl_2_, 1% polyethylene glycol 6000) [47,48] for 1 hour to minimize the effects of any endogenous hormones that might mask the effects of 20E addition. One lobe from each pair of glands was then transferred to fresh TB1 containing 0.5 μM 20E (Sigma) while the sister lobe was transferred to fresh TB1 containing solvent (ethanol). As with the cells, all incubations were carried out for two or four hours and were performed at 22°C in triplicate.

### RNA isolation and hybridization to cDNA arrays

TRIzol reagent (Invitrogen) was used to isolate total RNA from both cells and glands according to the manufacturer's protocol. For each hormone treatment performed, RNA was isolated independently from three biological replicates. Quality and quantity of RNA was verified by spectrophotometry and the A260/A280 ratios were greater than 1.8. RNA extracted from glands was subjected to linear amplification using MessageAmp™ II aRNA Amplification Kit (Ambion). Labeling and hybridization to microarrays was carried out as described on the CDMC website (http://www.flyarrays.com and Neal et al. 2003 [49]). Briefly, 2 μg of amplified RNA from glands or 80 μg of total RNA from cells was reverse-transcribed with SuperScriptII (Invitrogen) in the presence of cyanine (Cy) dye coupled nucleotides. After isopropanol precipitation of the labeled cDNA, Cy5 labeled cDNA generated from experimental samples was mixed with Cy3 labeled cDNA from the respective untreated control sample and hybridized to the 12k_v1 cDNA microarray from the Canadian *Drosophila *Microarray Centre (CDMC). All downstream processing and hybridization steps were performed exactly as previously described [49,50].

### Microarray data acquisition, normalization and analysis

A ScanArray 4000 laser scanner (Perkin Elmer) was used to acquire 16-bit TIFF images of the hybridized arrays that were subsequently analyzed with QuantArray v3.0 software (Perkin Elmer). Quantification data files and their associated images were loaded into GeneTraffic (GT) DUO (Iobion Informatics/Stratagene) where spots with raw intensities less than twice the average background or less than 128 fluorescence units were excluded from further analysis. Normalization and other analysis was performed following the guidelines outlined in Neal et al., 2003 and Neal and Westwood 2006 [49,50]. Briefly, normalization of the data was performed in GT using the subgrid Lowess algorithm with a 20% smoothing factor for all experiments to correct for systematic differences in data collection. Normalized data from 3 replicate arrays was complied and imported into Excel (Mircosoft) for significance analysis. At this point, genes represented by less than 2/3 valid spots were removed. The Significance Analysis of Microarrays (SAM) package was used to identify genes whose expression significantly differs between samples with a false discovery rate (FDR) of less than 5% [51]. Lists of genes found significant by SAM that also showed a change of at least 1.5 fold a coefficient of variation ≤1 were generated in GT DUO for each of the treatments under investigation. Hierarchical clusters were generated using the Pearson uncentered distance metric in MeV [52]. The List Functions tool available on the CDMC web site (http://www.flyarrays.com) was used to compare lists of 20E-responsive genes identified in our study with those previously identified. Data for this study can be found in Gene Expression Omnibus (GEO; http://www.ncbi.nlm.nih.gov/geo) under the accession GSE23928.

### Quantitative RT-PCR

One microgram of total RNA isolated from hormone-treated cells or salivary glands was treated with DNase I (Fermentas) to remove contaminating genomic DNA and then reverse-transcribed with SuperScript II reverse-transcriptase (Invitrogen) from anchor oligo dT primers (Invitrogen). The resulting cDNA was treated with RNase H to remove the RNA component of the cDNA-RNA hybrids prior to PCR. The following primers were used for amplification: Act5Cf: GTG CCC ATC TAC GAG GGT TA, Act5Cr: GCC ATC TCC TGC TCA AAG TC, E75f: CTG CCA GTA TTT CCA GTC, E75r: GGA CAA TGT GGG ATA CCT, E74f: CTA TTC ATG GGC GTT AGT, E74r: GAC AGT TGA AAG GTC ATT AG, Brf: ACA ACA ACA GCC CCG ACT T, Brr: GCT TGT CGC TGA TGG AGA TT, CG5346f: CGC TAG TTC AGG TGT ATC T, CG5346r: ACT TGT GCT CGC TAT ATC T, EcR-RAf: CAT AGG AGT CTT CAG TCT ACA, EcR-RAr: AGA TGG GGA TAG GGA TAC, EcR-RBf: CAT GGA TAC TTG TGG ATT AG, EcR-RBr: CTG GCA GTT GGT CTA TGT, EcR-RCf: TTG TGG ATT AGT AGC AGA AC, EcR-RCr: ACA CTT TCG CCT CAT GTA, EcR-RD/Ef: GCT ATA AAG ACA GGG AGA AC, EcR-RD/Er: GCA AAA TAT GGC TAG GTA AG, EcRf: GGA GAT TCT TGA CCT TAT GA, EcRr: TTT GTA AAC GCT GGT AGA C, USPf: GCG ATG AAA CTG GAG TAG, USPr: TGT AGG GTA TAA GGG ATA GAG. Triplicate qPCR reactions were performed with SYBR qPCR universal kit (KAPA) in a MX4000 qPCR instrument (Stratagene) under the following cycling parameters: 95°C for 10min followed by 40 cycles of 95° for 15s, 55°C for 25s and 72°C for 40s. A dissociation curve was plotted at the end of each run as a quality control for non-specific amplification products. For each gene the fold change ratio (relative to an untreated control) was normalized to Act5C mRNA level and calculated using the Pfaffl (ΔΔ C_t_) method [53]. The results presented are calculated from the mean fold change of two independent biological replicates.

### Analysis of functional classes

Following the hierarchical cluster analysis, genes with similar expression profiles were examined to see if their products shared any functional annotations. The CDMC Lookup tool available at http://www.flyarrays.com was used to obtain their corresponding LocusLink ID (now called EntrezGene ID) for use in DAVID [54,55]. DAVID assesses functional annotation associated with groups of genes for enrichment over the background represented by all genes on the 12k array. Annotation terms/keywords with EASE scores of ≤0.05 were taken as significantly enriched in a group of related genes and used to assign functional annotation to the group.

## Authors' contributions

SEG participated in the design of the study, carried out the hormone treatments, RNA isolations, microarray experiments, data analysis including analysis of functional classes, and qRTPCR experiments, and drafted the manuscript. SJN and ASK participated in the design of the study and participated in pilot experiments. JTW conceived of the study, participated in its design and helped to draft the manuscript. All authors read and approved the final manuscript.

## Supplementary Material

Additional file 1 **20E-responsive genes in Kc167 cells after 2 and 4 hours of exposure to 0.5 μM 20E**. Of the 27 genes induced by 0.5 μM 20E after 2 hours, one third are no longer up-regulated after 4 hours (group A) while the rest show similar levels of induction at both time points (group B). Many genes (51) are only significantly up-regulated after 4 hours of exposure to the hormone (group C). Likewise, genes that are repressed by 0.5 μM 20E are only detectable after 4 hours of treatment (group D). q-values determined by SAM represent the lowest false discover rate at which that gene is considered significant (see the Table 1 legend for details of q-values). A missing fold change value means that gene was either not represented by at least 2/3 spots and/or did not exhibit a coefficient of variation of at least 1 for that treatment across replicate arrays. Click here for file

Additional file 2 **Extended version of Table 1**. See Table 1 legend for details. Click here for file

Additional file 3 **qRT-PCR confirmation of microarray data**. Relative induction of selected genes identified by microarray analysis in Kc cells and salivary glands by either 0.5 μM 20E or 0.0625 μM PoA was confirmed by qRT-PCR relative to untreated Kc cells or salivary glands, respectively. Click here for file

Additional file 4 **Genes identified as part of the transcriptional response to either 20E or PoA**. Genes that exhibit a 1.5 fold change or greater to 0.0625 μM PoA in Kc167 cells or to either 10 μM 20E (20E high) or 0.5 μM 20E (20E) in Kc cells or to 0.5 μM 20E in salivary glands (glands) are listed. q-values determined by SAM represent the significance of fold change reported (see the Table 1 legend for details of q-values). Click here for file

Additional file 5 **Genes that respond to 20E in salivary glands and in *Drosophila *organ culture**. Genes identified as primary 20E-response genes by Beckstead and colleagues [13] are highlighted in grey. Click here for file

Additional file 6 **Salivary gland-specific 20E responsive genes**. These genes were identified as part of the response to 20E in salivary glands but not in whole organ culture [13]. Click here for file
