# Supplementary figures and images for: Genome-wide examination of the transcriptional response to ecdysteroids 20-hydroxyecdysone and ponasterone A in Drosophila melanogaster
Source: BMC Genomics. 2011 Sep 29;12:475. doi: 10.1186/1471-2164-12-475 (PMC3228561; doi:10.1186/1471-2164-12-475)

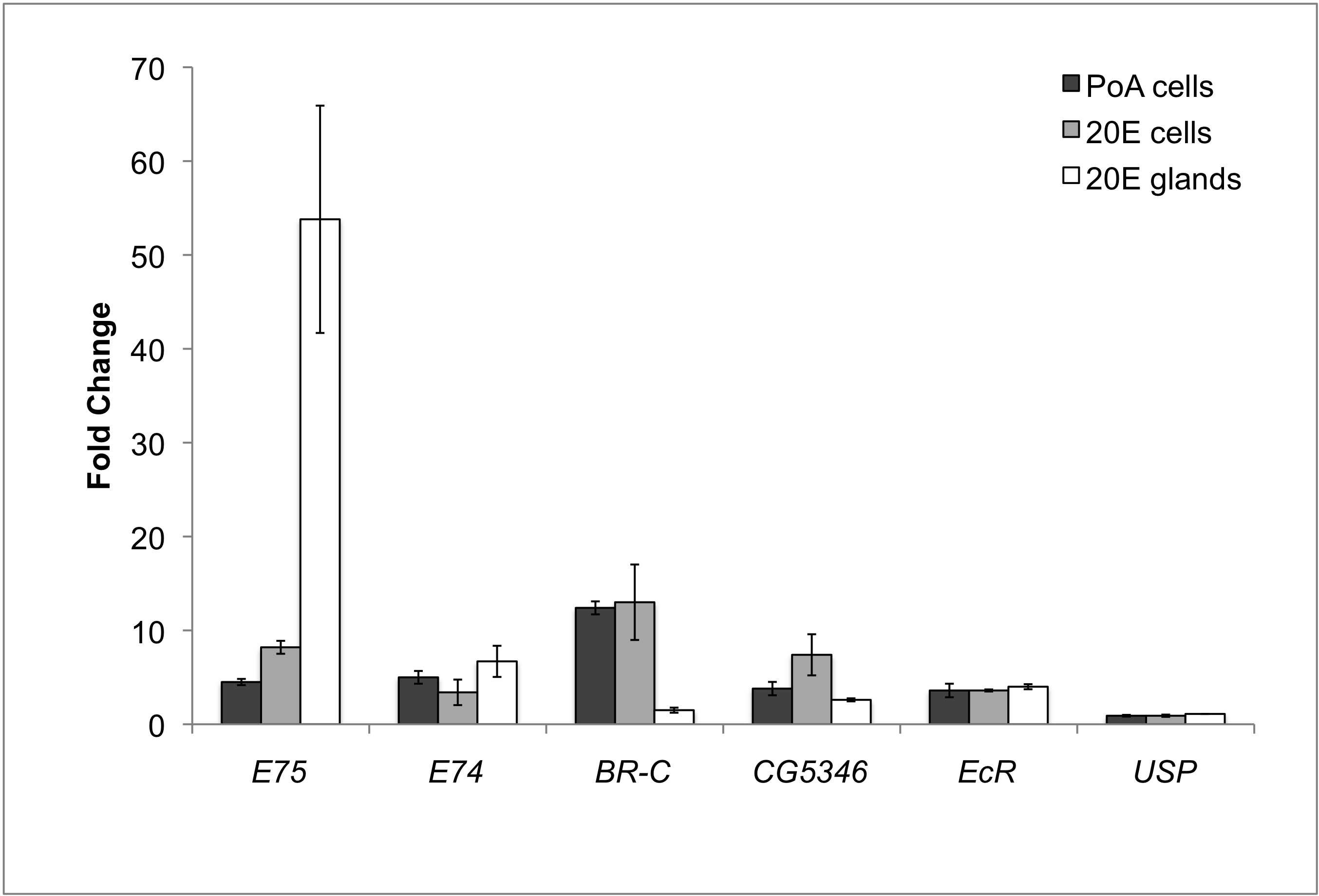

Supplement: Additional file 3 — qRT-PCR confirmation of microarray data. Relative induction of selected genes identified by microarray analysis in Kc cells and salivary glands by either 0.5 μM 20E or 0.0625 μM PoA was confirmed by qRT-PCR relative to untreated Kc cells or salivary glands, respectively. [file 1471-2164-12-475-S3.DOC]
