# Supplementary material for: Genome-wide examination of the transcriptional response to ecdysteroids 20-hydroxyecdysone and ponasterone A in Drosophila melanogaster
Source: BMC Genomics. 2011 Sep 29;12:475. doi: 10.1186/1471-2164-12-475 (PMC3228561; doi:10.1186/1471-2164-12-475)
Supplement: Additional file 5 — Genes that respond to 20E in salivary glands and in Drosophila organ culture. Genes identified as primary 20E-response genes by Beckstead and colleagues [13] are highlighted in grey. [file 1471-2164-12-475-S5.DOC]

| CG number | salivary glands 20E fold change | salivary glands q value (%) | Beckstead et al. (2005)- organ culture 20E fold change | Beckstead et al. (2005)- organ culture q value (%) | GenBank Accession | Gene Name |
| --- | --- | --- | --- | --- | --- | --- |
| CG32180 | 5.94 | 0.00 | 12.56 | 1.29 | AE003523 | Ecdysone-induced protein 74EF |
| CG11170 | 5.12 | 0.00 | 1.56 | 2.00 | AE003456 | CG11170 |
| CG1210 | 3.61 | 0.00 | 1.47 | 3.80 | AA201214 | Protein kinase 61C |
| CG3897 | 3.48 | 0.00 | 1.40 | 5.35 | AA141175 | bloated tubules |
| CG7656 | 2.70 | 0.00 | 1.40 | 4.37 | AE003530 | CG7656 |
| CG9393 | 2.68 | 0.00 | 1.59 | 4.19 | AA141814 | CG9393 |
| CG3409 | 2.67 | 0.00 | 1.51 | 4.37 | AA141210 | CG3409 |
| CG8676 | 2.59 | 0.00 | 1.89 | 2.00 | AA440081 | Hormone receptor-like in 39 |
| CG4173 | 2.49 | 0.00 | 1.43 | 2.11 | AA202391 | Septin-2 |
| CG8705 | 2.42 | 0.00 | 1.53 | 1.13 | AA201950 | peanut |
| CG7806 | 2.41 | 0.40 | 1.33 | 7.03 | AA539405 | CG7806 |
| CG7717 | 2.40 | 0.00 | 1.33 | 5.35 | AA950070 | Mekk1 |
| CG8502 | 2.29 | 0.00 | 1.69 | 6.20 | AE003822 | Cuticular protein 49Ac |
| CG8954 | 2.15 | 0.00 | 1.43 | 3.47 | AA202382 | Smg5 |
| CG9772 | 2.12 | 1.09 | 1.45 | 1.29 | AA246762 | CG9772 |
| CG4427 | 2.12 | 0.67 | 5.92 | 0.94 | AE003589 | cabut |
| CG10916 | 2.08 | 0.00 | 1.38 | 1.13 | AE003800 | CG10916 |
| CG1765 | 2.03 | 0.00 | 1.51 | 2.79 | AA201657 | Ecdysone receptor |
| CG11275 | 1.99 | 0.00 | 1.66 | 1.97 | AA695707 | CG11275 |
| CG8839 | 1.99 | 0.00 | 1.36 | 2.79 | AA246375 | CG8839 |
| CG31363 | 1.92 | 0.00 | 1.32 | 7.03 | AA263967 | Jupiter |
| CG3714 | 1.84 | 0.24 | 2.35 | 4.81 | AA567944 | CG3714 |
| CG3671 | 1.83 | 0.00 | 1.45 | 3.47 | AA820638 | Malvolio |
| CG11089 | 1.80 | 0.00 | 1.65 | 5.37 | AA438421 | CG11089 |
| CG12773 | 1.79 | 1.48 | 1.40 | 2.55 | AA440635 | CG12773 |
| CG4373 | 1.79 | 0.00 | 1.65 | 3.47 | AE003458 | Cyp6d2 |
| CG16708 | 1.77 | 0.00 | 1.70 | 2.00 | AA202475 | CG16708 |
| CG9934 | 1.77 | 3.35 | 1.53 | 2.55 | AA820898 | CG9934 |
| CG12749 | 1.74 | 1.09 | 1.40 | 3.45 | AA201135 | Heterogeneous nuclear ribonucleoprotein at 87F |
| CG3637 | 1.74 | 0.00 | 1.86 | 3.47 | AA950022 | Cortactin |
| CG5134 | 1.73 | 0.00 | 1.53 | 5.47 | AA264107 | Mediator complex subunit 9 |
| CG33991 | 1.71 | 0.67 | 1.42 | 6.77 | AA202853 | nuclear fallout |
| CG10082 | 1.68 | 0.40 | 1.46 | 3.02 | AA201509 | CG10082 |
| CG8091 | 1.66 | 0.00 | 1.57 | 3.80 | AA202030 | Nedd2-like caspase |
| CG6891 | 1.66 | 0.40 | 1.62 | 6.01 | AE003510 | CG6891 |
| CG8024 | 1.66 | 1.48 | 1.75 | 1.29 | AA201995 | lightoid |
| CG8127 | 1.64 | 0.00 | 2.80 | 0.94 | AA141333 | Ecdysone-induced protein 75B |
| CG8946 | 1.64 | 1.09 | 1.76 | 5.37 | AA201902 | Sphingosine-1-phosphate lyase |
| CG7337 | 1.63 | 0.40 | 1.69 | 3.47 | AA391890 | CG7337 |
| CG31919 | 1.62 | 1.48 | 1.42 | 8.95 | AA695044 | CG31919 |
| CG12004 | 1.61 | 1.09 | 1.43 | 6.20 | AA433279 | CG12004 |
| CG17161 | 1.61 | 0.40 | 1.32 | 6.01 | AA141959 | grapes |
| CG10719 | 1.57 | 1.48 | 2.94 | 0.94 | AA438467 | brain tumor |
| CG6264 | 1.55 | 4.09 | 1.34 | 3.95 | AA816705 | Bestrophin 1 |
| CG6287 | 1.55 | 0.91 | 1.56 | 5.37 | AA264623 | CG6287 |
| CG18769 | 1.55 | 0.67 | 1.40 | 5.37 | AA264886 | CG18769 |
| CG10842 | 1.53 | 4.98 | 1.82 | 3.47 | AE003834 | Cytochrome P450-4p1 |
| CG11880 | 1.52 | 0.00 | 1.57 | 9.60 | AA697340 | CG11880 |
| CG3962 | 1.52 | 0.40 | 1.82 | 2.00 | AE003716 | Keap1 |
| CG4860 | 1.51 | 0.00 | 1.42 | 1.29 | AA697101 | CG4860 |
| CG2718 | 1.50 | 2.99 | 1.86 | 6.01 | AA390709 | Glutamine synthetase 1 |
| CG14029 | 1.34 | 0.00 | 3.54 | 2.11 | AC008324 | vrille |
