# Supplementary material for: Genome-wide examination of the transcriptional response to ecdysteroids 20-hydroxyecdysone and ponasterone A in Drosophila melanogaster
Source: BMC Genomics. 2011 Sep 29;12:475. doi: 10.1186/1471-2164-12-475 (PMC3228561; doi:10.1186/1471-2164-12-475)
Supplement: Additional file 6 — Salivary gland-specific 20E responsive genes. These genes were identified as part of the response to 20E in salivary glands but not in whole organ culture [13]. [file 1471-2164-12-475-S6.DOC]

| Gene ID | Fold Change | q value (%) | GenBank accession | Gene Name | GO Molecular Function | GO Biological Process |
| --- | --- | --- | --- | --- | --- | --- |
| SD05494 | 3.07 | 0.00 | N/A |  |  |  |
| CG32137 | 2.91 | 0.00 | AE003536 | CG32137 | cytoskeletal protein binding | cell cycle |
| CG3766 | 2.62 | 0.00 | AA817247 | scattered | cation binding | cell development |
| CG8677 | 2.60 | 0.00 | AA264511 | CG8677 | DNA binding | cellular metabolism |
| CG9487 | 2.45 | 0.00 | AE003622 | - | protein binding |  |
| CG5582 | 2.43 | 3.35 | AE003522 | cln3 |  | cell-cell signaling |
| CG8448 | 2.39 | 0.67 | AA391802 | mrj | heat shock protein binding | cellular metabolism |
| CG9705 | 2.36 | 0.00 | AA201757 | CG9705 | DNA binding | cellular metabolism |
| CG6606 | 2.35 | 0.00 | AA264704 | Rab11 interacting protein | protein binding |  |
| CG2762 | 2.30 | 0.00 | AA140715 | u-shaped | DNA binding | anterior/posterior pattern formation |
| CG13698 | 2.27 | 0.00 | AE003521 | CG13698 |  |  |
| CG1756 | 2.23 | 0.00 | AE003486 | CG1756 | alpha-type channel activity | cell-cell signaling |
| CG6424 | 2.23 | 0.00 | AA696359 | CG6424 | protein binding |  |
| CG9610 | 2.08 | 0.00 | AA940837 | Pox meso | DNA binding | cell proliferation |
| CG7400 | 2.08 | 0.00 | AA698552 | Fatty acid (long chain) transport protein | fatty acid transporter activity | cellular metabolism |
| CG5620 | 2.07 | 0.91 | AA539907 | approximated | metal ion binding |  |
| CG32146 | 2.07 | 0.24 | AA142268 | dally-like | protein binding | blastoderm segmentation |
| RE61907 | 2.07 | 0.00 | N/A |  |  |  |
| CG5467 | 2.04 | 0.91 | AE003757 | CG5467 |  |  |
| CG32306 | 2.01 | 1.09 | AA140663 | CG32306 |  |  |
| CG11652 | 2.01 | 0.00 | AA264244 | CG11652 | protein binding | defense response |
| CG33324 | 1.95 | 0.00 | AE003675 | CG33324 |  |  |
| CG31729 | 1.83 | 0.00 | AA392479 | CG31729 | ATPase activity | establishment of localization |
| CG10173 | 1.80 | 0.40 | AE003563 | Bestrophin 2 | protein binding | establishment of localization |
| CG4043 | 1.80 | 0.00 | AA695032 | Rrp46 | RNA binding | cellular metabolism |
| CG15365 | 1.77 | 0.00 | AE003446 | CG15365 |  |  |
| CG10527 | 1.75 | 1.48 | AA802489 | CG10527 | transferase activity |  |
| CG6579 | 1.73 | 0.00 | AE003635 | CG6579 |  |  |
| CG7594 | 1.73 | 0.67 | AE003530 | Eig71Eh |  |  |
| CG4040 | 1.69 | 1.09 | AA536389 | CG4040 | protein binding |  |
| CG10833 | 1.68 | 0.00 | AA697564 | Cyp28d1 | monooxygenase activity | cellular metabolism |
| CG7207 | 1.66 | 1.09 | AA941020 | CG7207 | transferase activity | cellular metabolism |
| CG18490 | 1.65 | 0.40 | AE003545 | CG18490 |  |  |
| CG7340 | 1.60 | 0.00 | AA439330 | granny smith | cation binding | cellular metabolism |
| CG7228 | 1.58 | 0.00 | AA141088 | peste | transmembrane receptor activity | cell activation |
| CG2928 | 1.57 | 0.00 | AA698461 | Rhythmically expressed gene 5 |  | circadian rhythm |
| CG10863 | 1.56 | 1.98 | AA264709 | CG10863 | oxidoreductase activity |  |
| CG6692 | 1.55 | 0.00 | AA141403 | Cysteine proteinase-1 | peptidase activity | catabolism |
| CG9381 | 1.55 | 4.09 | AA140662 | murashka | cation binding | cellular metabolism |
| CG12085 | 1.54 | 1.09 | AA201432 | poly U binding factor 68kD | RNA binding | cell cycle |
| GH11072 | 1.53 | 0.00 | N/A |  |  |  |
| CG9901 | 1.52 | 1.09 | AA440440 | Actin-related protein 14D | cytoskeletal protein binding | cell organization and biogenesis |
| LP01301 | 1.52 | 0.00 | N/A |  |  |  |
| CG6842 | 1.51 | 1.48 | AA736192 | Vacuolar protein sorting 4 | hydrolase activity | cell organization and biogenesis |
| CG15009 | 1.51 | 0.00 | AA246942 | Ecdysone-inducible gene L2 |  | cell adhesion |
| CG5346 | 1.51 | 1.09 | AE003739 | CG5346 | cation binding |  |
